# Hepatic Fat Quantification with the Multi-Material Decomposition Algorithm by Using Low-Dose Non-Contrast Material-Enhanced Dual-Energy Computed Tomography in a Prospectively Enrolled Cohort

**DOI:** 10.3390/medicina58101459

**Published:** 2022-10-15

**Authors:** Seung Baek Hong, Nam Kyung Lee, Suk Kim, Kyunga Um, Keunyoung Kim, In Joo Kim

**Affiliations:** 1 Department of Radiology, Biomedical Research Institute, Pusan National University Hospital, Pusan National University School of Medicine, Pusan 46241, Korea; 2 General Electronics (GE) Healthcare Korea, Seoul 04637, Korea; 3 Department of Nuclear Medicine and Biomedical Research Institute, Pusan National University Hospital, Pusan 46241, Korea; 4 Division of Endocrinology and Metabolism, Department of Internal Medicine, Biomedical Research Institute, Pusan National University Hospital, Pusan 46241, Korea

**Keywords:** computed tomography, magnetic resonance image, hepatic fat, radiation, diagnostic performance

## Abstract

The early diagnosis of hepatic steatosis is important. No study has assessed hepatic fat quantification by using low-dose dual-energy computed tomography (CT). We assessed the accuracy of hepatic fat quantification using the multi-material decomposition (MMD) algorithm with low-dose non-contrast material-enhanced dual-energy CT. We retrospectively reviewed 33 prospectively enrolled patients who had undergone low-dose non-contrast material-enhanced dual-energy CT and magnetic resonance image (MRI) proton density fat fraction (PDFF) on the same day. Percentage fat volume fraction (FVF) images were generated using the MMD algorithm on the low-dose dual-energy CT data. We assessed the correlation between FVFs and MRI-PDFFs by using Spearman’s rank correlation. With a 5% cutoff value of MRI-PDFF for fatty liver, a receiver operating characteristic (ROC) curve analysis was performed to identify the optimal criteria of FVF for diagnosing fatty liver. CTDIvol of CT was 2.94 mGy. FVF showed a strong correlation with MRI-PDFF (r = 0.756). The ROC curve analysis demonstrated that FVF ≥ 4.61% was the optimal cutoff for fatty liver. With this cutoff value for diagnosing the fatty liver on low-dose dual-energy CT, the sensitivity, specificity, and area under the curve were 90%, 100%, and 0.987, respectively. The MMD algorithm using low-dose non-contrast material-enhanced dual-energy CT is feasible for quantifying hepatic fat.

## 1. Introduction

Hepatic steatosis (HS) is a major cause of liver disease and is associated with various metabolic comorbidities such as obesity, hypertension, hyperlipidemia, and type 2 diabetes. Its reported prevalence is 25–32% in the population [1]. Among patients with HS, those with non-alcoholic fatty liver disease (NAFLD), which is the most common chronic liver disease, present the absence of specific causes (e.g., viral hepatitis, alcohol, or medication) [2]. NAFLD is projected to become the leading indication for liver transplantation in the United States over the next decade [3].

The early diagnosis of HS is important. For example, a subgroup of patients with NAFLD can develop non-alcoholic steatohepatitis (NASH), which is characterized by pathological hepatocyte damage and associated with hepatic fibrosis [4]. Therefore, NAFLD can cause liver-specific mortality and morbidity (e.g., hepatocellular carcinoma, decompensated liver, and cirrhosis) [5,6].

Liver biopsy is still the gold standard for diagnosing NAFLD (e.g., simple HS, inflammation, ballooning, and fibrosis). However, it has several limitations such as invasiveness, observer dependency, poor acceptability, and sampling errors [7,8,9,10]. Therefore, noninvasive tools have been developed, including ultrasound (US), computed tomography (CT), and magnetic resonance (MR) imaging (MRI), for diagnosing the spectrum of NAFLD [11,12].

Among the aforementioned imaging modalities, unenhanced CT can be a semiquantitative tool for diagnosing HS with an attenuation value [13]. Although CT is convenient tool, it exhibits relatively low sensitivity for diagnosing HS in patients with mild HS (≥5% for pathological results) [14,15]. Moreover, contrast-enhanced CT presented inferior results for predicting HS due to confounding factors, such as iodinated contrast media [13].

Recently, hepatic fat quantification using dual-energy CT has been increasing and has provided reliable results. In particular, the multi-material decomposition (MMD) algorithm was used for hepatic fat quantification in dual-energy CT. In the MMD algorithm, the degree of HS is presented as the hepatic fat volume fraction (FVF) with multi-material decomposition, including hepatic fat. Several studies have demonstrated the feasibility of the MMD algorithm for assessing the FVF [16,17,18]. However, to our knowledge, no study has assessed hepatic FVF by using low-dose dual-energy CT.

Therefore, this study evaluated the feasibility of hepatic fat quantification using the MMD algorithm with low-dose unenhanced dual-energy CT.

## 2. Materials and Methods

The study cohort had been prospectively enrolled for another study (“Clinical study of effectiveness of DXA for the evaluation of body composition,” not published). This study was retrospectively reviewed and aimed to assess the accuracy of hepatic fat quantification with the MMD algorithm using low-dose dual-energy CT. The study was approved by our institutional review board (IRB No. 2208-011-117), and the requirement for informed consent was waived due to its retrospective nature.

### 2.1. Patients

**Figure 1:**
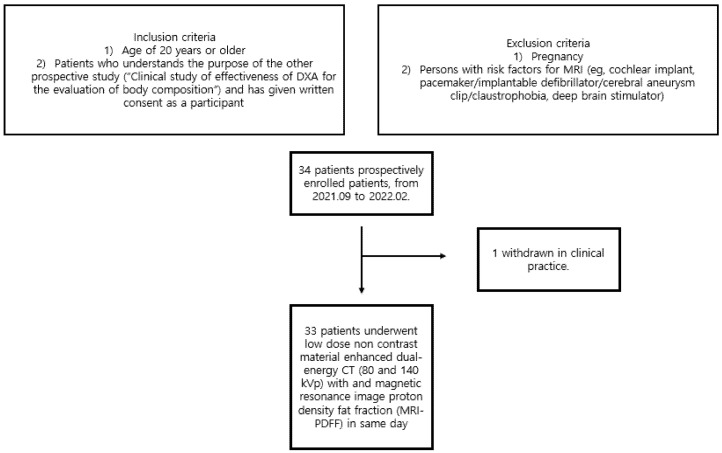
Flow diagram of the study population.

All patients were: (1) aged 20 years or older; (2) those who understood the purpose of the study (“Clinical study of effectiveness of DXA for the evaluation of body composition”) and provided written consent to participate. The exclusion criteria for the study participants were: (1) pregnancy; (2) patients with risk factors for MRI (e.g., cochlear implant, pacemaker/implantable defibrillator/cerebral aneurysm clip/claustrophobia, deep brain stimulator). Based on the aforementioned inclusion and exclusion criteria, 34 patients were prospectively enrolled from September 2021 to February 2022. Among these, one patient was excluded due to incidentally detected multiple hepatic masses on US examination in clinical practice. Therefore, 33 patients were enrolled and underwent low-dose unenhanced dual-energy CT (80 and 140 kVp) with MRI proton density fat fraction (MRI-PDFF) on the same day (Figure 1).

### 2.2. Imaging Technique

#### 2.2.1. Dual-Energy CT Acquisition

Low-dose unenhanced dual-energy CT was performed using a 256 slice CT scanner (Revolution APEX; GE Healthcare, Waukesha, Wis, USA). The scanning parameters were as follows: ultra-fast 80–140 kV switching; 145 mA; rotation time, 0.5 s (s); beam collimation, 40 mm; pitch, 1.375:1; reconstruction algorithm, standard kernel; slice thickness, 2.5 mm. The scanning parameters are listed in Table 1. Hepatic FVF images were reconstructed from low-dose unenhanced dual-energy datasets.

#### 2.2.2. MRI-PDFF

Multi-echo gradient echo sequences (ME Dixon) with inline reconstruction were performed using a 3.0 T MR scanner (Skyra; Siemens Healthineers). Six fractional echo magnitude images were acquired during a 13 s breath hold. Detailed parameters were as follows: flip angle, 4°; echo times (TE), 1.09, 2.46, 3.69, 4.92, 6.15, and 7.38 ms; repetition time (TR), 9 ms; section thickness, 3.5 mm; field of view, 332.5 × 380 mm; voxel size, 1.2 × 1.2 × 3.5 mm.

### 2.3. Data Analysis

One of the authors (S.K.; board-certified abdominal radiologist; 27 years of clinical experience), who did not participate in the assessment of FVF, placed the five regions of interest (ROIs) with 1000.00 mm^2^ of area on the liver right lobe in MRI-PDFF, avoiding the large vessel, and compiled the MR images with only the location of the measurement. The mean ROI value was calculated and recorded for each patient.

Hepatic FVF images with a 2.5-mm thickness were reconstructed using the MMD-based fat quantification algorithm. Using a gemstone spectral imaging volume viewer of advantage workstation (AW VolumeShare 7; GE Healthcare), FVF was independently assessed by two board-certified abdominal radiologists (N.K.L. and S.B.H.; 19 and 10 years of clinical experience, respectively). Two radiologists were provided with MR images containing information only on the locations measured during the previous MRI-PDFF procedure. According to the aforementioned MR images, each radiologist, who was blinded to the values of MRI-PDFF, placed five ROIs with 1000.00 mm^2^ of area on the right lobe of the liver for assessment of FVF, avoiding the large vessel. The mean ROI values of the ROIs were calculated for each patient.

The dose-length product (DLP) and volumetric CT dose index (CTDIvol) of low-dose dual-energy CT were reviewed along with dose reports in the PACS. The effective dose in millisieverts (mSv) was calculated as DLP × k (tissue conversion coefficient 0.015).

### 2.4. Statistical Analysis

We assessed the correlation between the mean FVFs assessed by the two radiologists and MRI-PDFFs using Spearman rank correlation. As described in a previous study, 5% in MRI-PDFF was considered the cutoff value for diagnosing fatty liver in this study [19,20]. A receiver operating characteristic (ROC) curve analysis was performed to identify the optimal criteria for FVF for diagnosing fatty liver. Additionally, we calculated the sensitivity, specificity, and area under the curve (AUC).

To assess FVF, we evaluated inter-observer agreement using intra-class correlation coefficients (ICCs). The agreement was defined as poor (ICC, 0–0.4); fair-to-good (ICC, 0.40–0.75); or excellent (ICC, >0.75). SPSS version 22 (IBM, Armonk, NY, USA) was used for all statistical analyses.

## 3. Results

### 3.1. Patient Demographics

Thirty-three prospective patients were enrolled, 17 of whom were male. The mean age was 46.5 years. The number of patients who demonstrated the MRI-PDFF < 5.0% was 23. Among 10 patients who presented a MRI-PDFF of ≥ 5.0%, five presented a MRI-PDFF of < 15.0%. The patient demographics are summarized in Table 2.

### 3.2. Radiation Dose Measurement in Low-Dose Dual-Energy CT

The CTDIvol of the low-dose dual-energy CT was 2.94 mGy. The mean ± standard deviation DLP was 87.75 ± 6.48 mGy-cm. The mean ± standard deviation effective dose of low-dose dual-energy CT was 1.3 ± 0.1 mSv.

### 3.3. Inter-Observer Agreement

For the mean FV assessed by the two radiologists, we evaluated the inter-observer agreement using ICCs. The inter-observer agreement for assessing FVF was excellent. The ICC was 0.99.

### 3.4. Correlation of FVF with MRI-PDFF

**Figure 2:**
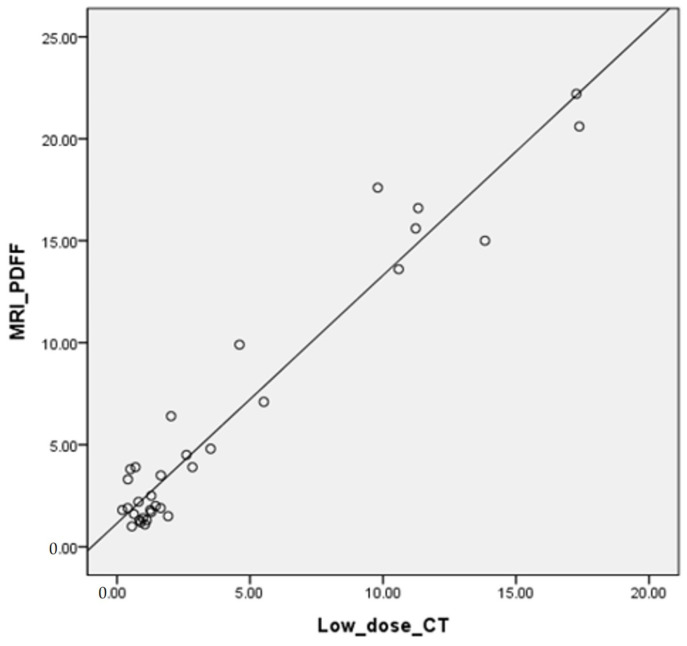
Correlation of FVF with MRI-PDFF.

With reference to MRI-PDFF, a correlation analysis was performed for FVF on low-dose dual-energy CT. The FVF and MRI-PDFF showed a strong correlation (r = 0.756; *p* < 0.001) (Figure 2).

### 3.5. ROC Curve Analysis of FVF for Diagnosing Fatty Liver

**Figure 3:**
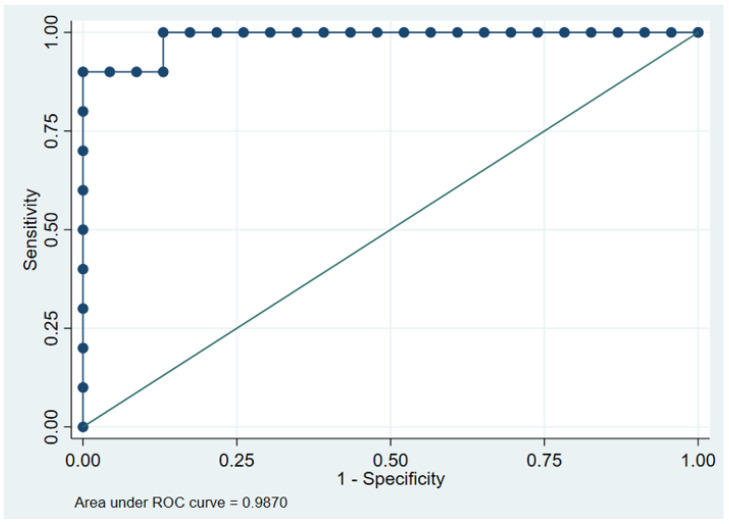
Receiver operating characteristics curve analysis of FVF for diagnosing fatty liver.

**Figure 4:**
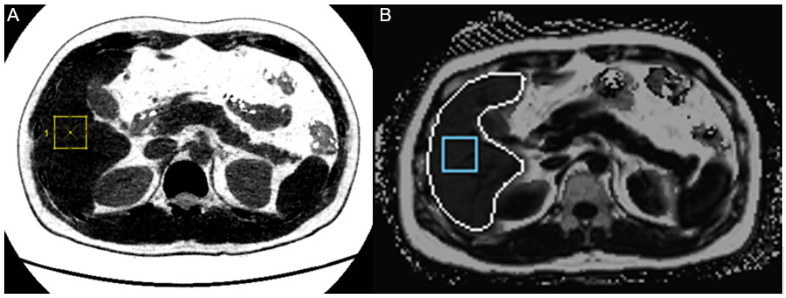
An example of fat quantification using (**A**) an FVF map and (**B**) MRI-PDFF in a 33-year-old man with fatty liver (MRI-PDFF = 6.5%). The FVF map yielded a value of 5.2%.

As aforementioned, 5% in MRI-PDFF was considered the cutoff value for diagnosing fatty liver in this study. The ROC curve analysis demonstrated a FVF of ≥ 4.61% as the optimal cutoff for diagnosing fatty liver. The AUC for differentiating normal liver from fatty liver was 0.987 (95% CI, 0.958–1.000) (Figure 3). With this FVF cutoff value for diagnosing fatty liver on low-dose dual-energy CT, the sensitivity and specificity were 90% and 100%, respectively (Figure 4).

## 4. Discussion

Various noninvasive tools including US, CT, and MRI have recently been implemented for diagnosing HS [11,12]. In this study, we assessed the feasibility of unenhanced dual-energy CT with a remarkably low radiation dose (CTDIvol, 2.94 mGy; mean effective dose, 1.3 ± 0.1 mSv) for assessing HS. With reference to MRI-PDFF, FVF on low-dose dual-energy CT and MRI-PDFF showed a strong correlation (r = 0.756). Furthermore, with an FVF cutoff value of ≥ 4.61%, low-dose unenhanced dual-energy CT demonstrated the excellent diagnostic performance for diagnosis of the fatty liver (AUC, 0.987; sensitivity, 90%; specificity, 100%).

The FVF cutoff value of ≥4.61% for predicting the HS identified in our results was in line with the results of a previous study by Hyodo et al. [17]. Although the reference standard of that study was the histological result, the authors also demonstrated that an FVF cutoff value of 4.6% could discriminate grade 0 histological steatosis from grades 1 to 3 in a study employing dual-energy CT using the MMD algorithm. They also reported its excellent diagnostic performance for discriminating grade 0 histological steatosis from grades 1 to 3 (AUC, 0.88; sensitivity, 82%; specificity, 100%). This diagnostic performance was also in line with our results. However, the radiation dose in our study was lower (CTDIvol, 2.94 mGy vs. 15.64 mGy; mean DLP, 87.75 mGy-cm vs. 533 mGy-cm).

Several studies have demonstrated the good diagnostic performance of dual-energy CT with the MMD algorithm for hepatic fat quantification [16,17]. These studies employed multiphase contrast-enhanced dual-energy CT without reducing the radiation dose. Although our study was performed using only unenhanced CT, none of the aforementioned studies demonstrated a significant difference in the FVF between the unenhanced CT phase and other contrast-enhanced scanning phases.

Optimal image acquisition with radiation dose reduction is important. However, in practice, unenhanced low-dose CT for abdominal organs is not routinely performed. CT images acquired using a low radiation dose cannot guarantee an image quality with acceptable noise. However, the recently developed deep-learning-based reconstruction is expected to become a key method for low-dose abdominal CT with acceptable image quality [21,22]. Generally, triple-phase contrast-enhanced CT, which is routinely used for the assessment of hepatic lesions, includes unenhanced CT. Therefore, unenhanced CT with low-dose dual-energy CT is a promising tool for hepatic fat quantification in routine practice.

US and MRI are radiation-free imaging modalities used for predicting HS in routine practice. US is a cost-effective tool for predicting HS severity. In conventional US, the severity of HS is assessed using subjective sonographic imaging patterns. Moreover, the severity of HS is stratified into four grades (e.g., absent, mild, moderate, severe) [23,24]. Conventional US has presented lower diagnostic sensitivity (50–62%) for predicting HS (≥ 5.0%) [12]. MRI is a more accurate modality for assessing HS than CT but is too expensive to deploy to the general HS population [25]. CT performed with a low radiation dose may be an option for imaging modalities in the workup for HS in the general HS population.

Our study had several limitations. First, our results were derived from a retrospective review. However, the study cohort enrollment was prospectively performed. Second, we included a relatively small cohort. Therefore, further prospective studies with larger cohorts are warranted. Third, there was no condition to exclude the patients with liver cancer, hepatic hemangioma, and other liver tumors. A total of 34 patients were prospectively enrolled from September 2021 to February 2022. Among these, one patient was excluded due to incidentally detected multiple hepatic masses on US examination in clinical practice. In a retrospective review for the final included 33 patients none had hepatic malignancy. Finally, the ROI was only located in the right lobe of the liver in the data analysis, as the purpose of our study was assessing the feasibility of the multi-material decomposition algorithm by using low-dose non-contrast material-enhanced dual-energy computed tomography with the reference standard as MRI-PDFF. For the inhomogeneity of fatty liver, our method had a limitation. To somewhat tackle this limitation, we placed the five ROIs on the liver right lobe in the data analysis.

## 5. Conclusions

In conclusion, low-dose unenhanced dual-energy CT with the MMD algorithm is a feasible tool for hepatic fat quantification. The MMD algorithm using low-dose dual-energy CT can be used for hepatic fat quantification in routine practice, with decreased ionizing radiation.

## Figures and Tables

**Table 1:** Protocol of the low-dose dual-energy CT.

Parameter	Values
Tube voltage (kV)	80–140
Tube current (mAs)	145
Rotation time (second)	0.5
Beam collimation (mm)	40
Pitch	1.375:1
Slice thickness (mm)	2.5

**Table 2:** The characteristics of the included patients.

	Data
No. of patients	33
Male/Female	17/16
Age *	46.5 (13.2)
MRI PDFF	
MRI PDFF < 5.0%	23
5.0% ≤ MRI PDFF < 15.0%	5
15.0% ≤ MRI PDFF	5

MRI PDFF, magnetic resonance image proton density fat fraction. * Data are mean ± standard deviation.

## Data Availability

The data that support the findings of this study are available on request to the corresponding author. The data are not publicly available due to privacy issues.
